# The Cost-Effectiveness of Emergency Hormonal Contraception with Ulipristal Acetate versus Levonorgestrel for Minors in France

**DOI:** 10.1371/journal.pone.0138990

**Published:** 2015-09-30

**Authors:** Ramona Schmid

**Affiliations:** HRA Pharma, Paris, France; Women’s Hospital, School of Medicine, Zhejiang University, China. 310006, CHINA

## Abstract

**Objective:**

To evaluate the cost-effectiveness of ulipristal acetate and levonorgestrel in minors in France, and analyze whether it is worthwhile to provide ulipristal acetate to minors free of charge.

**Methods:**

The cost-effectiveness of two emergency contraceptive methods was compared based on a decision-analytical model. Pregnancy rates, outcomes of unintended pregnancies, and resource utilization were derived from the literature. Resources and their costs were considered until termination or a few days after delivery. Deterministic and probabilistic sensitivity analyses were performed.

**Results:**

The cost of an unintended pregnancy in a French minor is estimated to be 1,630 € (range 1,330 € – 1,803 €). Almost 4 million € (3.1 € – 13.7 € million) in unintended pregnancy spending in 2010 could have been saved by the use of ulipristal acetate instead of levonorgestrel. The incremental cost of ulipristal acetate compared to levonorgestrel is 3.30 € per intake, or 418 € per pregnancy avoided (intake within 72 hours). In the intake within 24 hours subgroup, ulipristal acetate was found to be more efficacious at a lower cost compared to levonorgestrel.

**Conclusions:**

Ulipristal acetate dominates levonorgestrel when taken within 24 hours after unprotected intercourse, i.e., it is more effective at a lower cost. When taken within 72 hours, ulipristal acetate is a cost- effective alternative to levonorgestrel, given that the cost of avoiding an additional pregnancy with ulipristal acetate is less than the average cost of these pregnancies. In the light of these findings, it is worthwhile to provide free access to minors.

## Introduction

Unintended pregnancy in teenagers is considered to be a public health concern in many countries. Giving birth while still developmentally a child is associated with social, psychological, and economic disadvantages for the mother, the child, and society [1–4]. In England, almost 55,000 adolescent pregnancies occurred in 1998 [1]. In the USA, 22% of 20-year olds have experienced a pregnancy in their teens [1]. In France, pregnancies in teens are comparatively less frequent. The pregnancy rate in girls aged below 18 years was estimated to be 2.4%, corresponding to approximately 16,000 unintended pregnancies per year [5]. Contraceptive use is crucial in decreasing unintended pregnancy rates. In France, teens below the age of 18 years (minors) have been able to obtain free emergency contraception anonymously in a pharmacy or from their school nurse since 2002 [6]. Moreover, the government recently adopted a law to ensure the availability of free regular contraception to minors [7]. Access to emergency contraception is important because it has the potential to decrease unintended pregnancy rates. Emergency contraception is used after unprotected sexual intercourse to prevent an unintended pregnancy. Levonorgestrel is used as emergency contraception and has been available without prescription for many years in most European countries and globally. In 2010, more than 400,000 units of emergency contraception were reimbursed by the social health insurance fund in France [8]. Almost 90% of these units were used by minors (362,273 intakes) [8].

Contraceptive methods have shown to be cost-saving versus no method [9–13]. Long-acting release contraceptive methods such as intrauterine systems are more cost-effective than short-acting methods such as oral contraception [10–17]. Yet more than 75% of French minors who use contraception choose oral contraception and the remainder mainly use the male condom [18]. Despite widespread contraceptive coverage, a French study has shown that 65% of unplanned pregnancies occurred among women using contraception [19]. The main reason identified for failure was misuse of the methods. Ulipristal acetate is a new chemical entity that has proven to be more efficacious than levonorgestrel as an emergency contraceptive [20]. Today, ulipristal acetate is reimbursed in France at a higher cost than levonorgestrel [21]. Since March 2013, ulipristal acetate has been available free to girls between 15 and 17 years of age [7]. A prescription is still required for ulipristal acetate, which considerably limits fast and easy access. Yet ulipristal acetate complies with the European guidelines criteria for a change in classification to non-prescription drug. If ulipristal acetate achieves non-prescription status, then ulipristal acetate will be available to minors free and anonymous, directly in the pharmacy. Due to the higher costs of ulipristal acetate compared to levonorgestrel, this could represent an important increase in drug expenditure for emergency contraception to minors in France, but result in savings elsewhere due to its higher efficacy (Table 1).

**Table 1:** Probabilities of pregnancy and pregnancy outcome.

Probabilities		Base case (%)	Range (%)		Ref.
			Min	Max	
**Probability of pregnancy**					
intake within 72 hours	ulipristal acetate	1.36	0.85	2.05	[20]*
	levonorgestrel	2.15	1.50	2.98	
intake within 24 hours	ulipristal acetate	0.85	0.28	1.99	
	levonorgestrel	2.50	1.41	4.09	
**Result of unintended pregnancy**					
delivery		27.70	8.50	28.70	Base case: [8] [22] min: [20] max: [8]
voluntary termination		68.80	72.30	71.30	
miscarriage		3.50	19.20	0.00	

* HRA Pharma internal data; the extreme values of the pregnancy rates are the 95% CI (Clopper Pearson method) (see: S1 File).

The cost-effectiveness of ulipristal acetate versus levonorgestrel has been shown in studies based in the UK [23], Spain [24], and the USA [25]. None of these analyses specifically considered delivery to minors. This study will compare the cost-effectiveness of ulipristal acetate versus levonorgestrel in minors in France with the objective of analyzing whether it is worthwhile to provide ulipristal acetate free to minors once it is available without prescription.

## Methods

**Fig 1:**
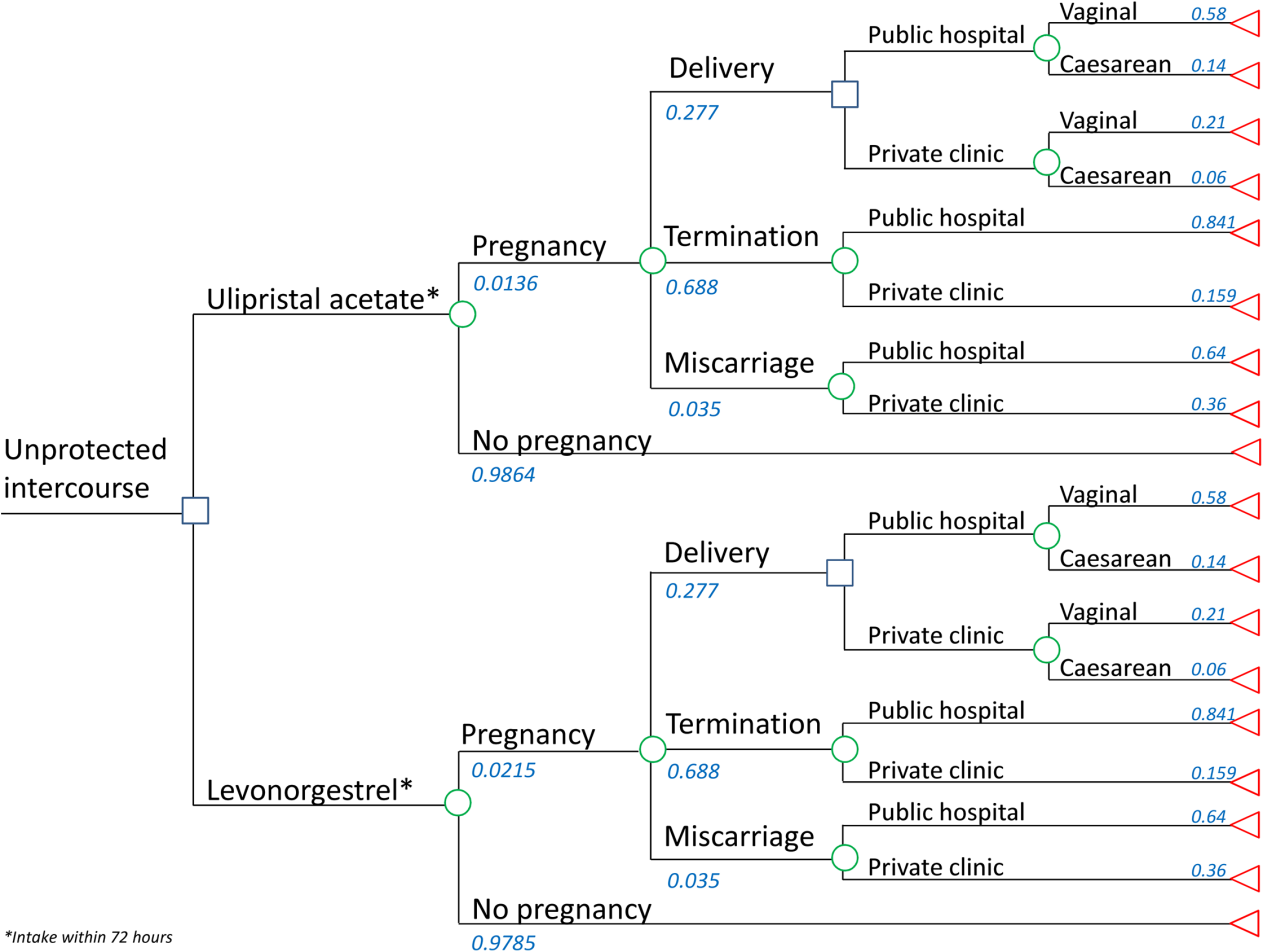
Decision analytic model.

A decision-analytical model was developed in TreeAge Pro 2013 (Fig 1). Intrauterine devices were not considered because they are not used as emergency contraception by young women in France, but only used as an option for women aged 35 years and older [8]. The target population is minors aged 15–17 who take emergency contraception. In the base case analysis, emergency contraception intake was within 72 hours, which corresponds to the recommended levonorgestrel dose and indication. The study’s time horizon was the period from unprotected intercourse until the moment termination occurred or, in the case of pregnancy carried to term, within 8 weeks after birth. In line with recommendations from the French guidelines for economic evaluations and cost-effectiveness analysis, a collective perspective was used, attempting to consider all the stakeholders concerned [26]. The collective perspective considers the cost of healthcare resources used whatever the source of funding. In the current analysis, such perspective includes compulsory and supplementary health insurance, as well as governmental funds. In line with the guidance, indirect costs were not included in the collective perspective. The model did not include transportation costs because of a lack of data, whilst costs for loss of productivity were not included because they were not considered relevant in a population of minors.

The efficacy of levonorgestrel versus ulipristal acetate was analyzed in a meta-analysis of two comparative randomized clinical trials [20]. Combining the two trials, 3,445 women taking emergency contraception within 24, 72, or 120 hours of unprotected intercourse were considered. It was shown that the risk of pregnancy with the use of ulipristal acetate after unprotected intercourse was significantly lower than the risk of pregnancy with the use of levonorgestrel for all time windows (Odds ratio 0.58 [0.33; 0.99] for intake within 72 hours). The risk was most greatly diminished in the first 24 hours after unprotected intercourse (Odds ratio 0.35 [0.11; 0.93]).

The consequences of unintended pregnancy considered in this analysis are voluntary termination, miscarriage, and term delivery. The proportions used for voluntary termination and delivery are based on French general population data. Data specific to French minors are used for the base case analysis. In 2009, 11,700 voluntary terminations and 4,700 term deliveries occurred in minors [8]. An estimated rate of miscarriage of 3.5% for young women has been added to these figures [22]. Thus, if 11,700 voluntary terminations plus 4,700 term deliveries are considered to represent 96.5% of that year’s unintended pregnancies, the total number of unintended pregnancies in French minors in 2009 can be estimated at 16,995. Using these numbers to express the outcome of unintended pregnancy by percentage, it can be estimated that 68.8% end in voluntary termination, 27.7% in delivery, and 3.5% in miscarriage. Voluntary termination is managed either medically or surgically, in a public hospital or private clinic. The respective proportions have been published specifically for French teens [27]. The majority of medical terminations are conducted in public hospitals (84.1%), and 12.8% are conducted in private hospitals [27]. Medical termination in France is also conducted in the outpatient setting in physician’s private practices. However, termination in a private practice is not allowed for minors by law. Therefore, it is assumed that these terminations (3.1%) [27] will be managed in a private clinic, leading to a figure of 15.9% of terminations managed in a clinic. Delivery at term includes outpatient and hospital care. Outpatient services for pre- and postnatal services are defined by law [28]. These services include obligatory medical examinations, preparation for delivery, and physician visits. In addition, families in France receive financial assistance in the seventh month of pregnancy to cover expenses linked to the upcoming birth [29]. Delivery takes place in a hospital by vaginal or Caesarean delivery. No data specific to minors has been defined, so the proportions observed in the general French population have been used in this study [30].

Only direct healthcare costs that are linked to the use of emergency contraception and to termination or term delivery after unintended pregnancy are considered in this analysis. The resources are valued using a production function according to the methodological guide for Economic Evaluation published by the French National Authority for Health [31]. Otherwise, tariffs are used for the valuation of resource use: the French government fixes the reimbursement rate for medical services based on pre-set rates that are negotiated and set annually. Doctors will be reimbursed by the social security system accordingly. As there are no differences in side effects between the two emergency contraception methods [20], the costs associated with side effects are not considered in this analysis. Drug prices are taken from the official French drug pricing database [21]. For the base case analysis, the price of Norlevo (HRA Pharma, France) is used because it is the most widely used levonorgestrel method currently available [32].

Termination costs are weighted per the location of the procedure. 84% of voluntary terminations are managed in a public hospital and 16% in a private clinic [27,30]. The costs of the diagnosis-related group 14Z08Z are taken into account in both sectors. The weighted average cost of a termination is estimated to be 477 €. Costs of delivery include hospital costs and costs of pre- and postnatal services for the delivery, as well as governmental assistance during pregnancy.

Hospital costs of term delivery are weighted per the method and location of delivery. 58% of births are vaginal delivery in a public hospital, and 21% are vaginal delivery in a private clinic. 14% of births are caesarean delivery in a public hospital, and 6% are caesarean delivery in a private clinic [30]. The costs of the following diagnostic related groups are taken in account in the public sector as well as in the private sector where available: caesarean delivery: 14C06A-D, 14C07A-D, 14C08A-D; vaginal delivery: 14Z10A-B and T, 14Z11A-B, 14Z12A-B, 14Z13A-D and T, 14Z14A-D and T, 14C03A-D. The weighted average hospital cost of delivery is estimated to be 2,608 €. In addition to hospital costs, pre- and postnatal care as described in the ministerial Decree is considered [28]. Table 2 indicates the services considered and their costs according to the tariff. Total costs of pre- and postnatal services are estimated to be 1,078 € per patient. The financial assistance from the government provided in the seventh month of pregnancy depends on family resources. It is assumed that minors do not have any revenue and hence benefit from the maximum financial assistance (923 €) [29]. Adding these costs to the hospital costs, the total cost of delivery is estimated to be 4,609 €.

**Table 2:** Pre- and postnatal care utilization and costs.

Outpatient costs							
	Number	Medical procedure code	Coeff.	Code*	Tariff, € (coeff x code)	Total costs, € (number x tariff)	Ref.
**Prenatal **							
Mandatory medical examinations							
clinical examination	7	-	1	CS	23.00	161.00	[33]
screening for HIV	1	388	60	B	16.20	16.20	[34] [35]
screening for Hepatitis B	1	4715	65	B	17.55	17.55	[34] [35]
proteinuria	7	1133	31	B	8.37	58.59	[34] [35]
glycosuria	7	2007	4	B	1.08	7.56	[34] [35]
toxoplasma serology	7	1430	60	B	16.20	113.40	[34] [35]
blood grouping	1	1140	35	B	9.45	9.45	[34] [35]
screening for syphilis	1	1326	20	B	5.40	5.40	[34] [35]
screening for rubella	1	1773	40	B	10.80	10.80	[34] [35]
Search for irregular antibodies	2	1141	45	B	12.15	24.30	[34] [35]
blood count (CBC/NFP)	1	1104	34	B	9.18	9.18	[34] [35]
detection of HBs antigen	1	4715	65	B	17.55	17.55	[34] [35]
Proposed medical examinations							
1. ultrasound	1	JQQM010	-	-	48.35	48.35	[36]
2. ultrasound	1	JQQM018	-	-	81.92	81.92	[36]
3. ultrasound	1	JQQM016	-	-	73.99	73.99	[36]
Childbirth preparation							
1. session	1	-	2.5	C	55.00	55.00	[37]
2–8. sessions	7	-	2	C	44.00	308.00	[37]
Total prenatal						1,018.24	
**Postnatal**	** **						
Clinical examination	1	-	1	CS	23.00	23.00	[33] [37]
Postnatal follow-up visits	2	-	1	SP	18.55	37.10	[33] [37]
Total postnatal						60.10	
**Total pre+ postnatal care**	** **					**1,078.34**	
Governmental assistance						923.08	[29]
Hospital costs						2,607.54	[30]
**Total cost of delivery**	** **					**4,608.96**	

* B, 0.27 €; C, 22.00 €; CS, 23.00 €; SP, 18.55 €. [33,34].

Hospital costs of miscarriage are weighted per the location of miscarriage. 64% of miscarriages are managed in a public hospital and 36% in a private clinic [30]. The costs of the diagnosis-related groups 14C05J and 14C05Z are taken into account in both sectors. The weighted average cost of miscarriage is estimated to be 728 € ([30], S2 File).

All costs and tariffs are cited as were used in 2010, except for the financial assistance. Although this assistance has existed for a longer time, no reference earlier than April 2013 has been identified (Table 3).

**Table 3:** Drug costs and cost of unintended pregnancy. Extreme values: ¤ hospital costs vary by +/-9% [29]; total costs of birth have been varied in the same range; § Norlevo (HRAPharma, France) and its generic price in case a generic drug will be launched;

	Base case	Min	Max	Ref.
Drug costs, €				
levonorgestrel	7.41	4.00	7.41	[21],§
ulipristal acetate	23.59	19.90	23.59	[21],*
Cost of pregnancy outcome, €				
cost of termination	476.65	396.23	557.08	Table 1, Table 2, [30]
cost of delivery¤	4,608.96	3,733.26	5,023.77	
cost of miscarriage	728.00	664.00	791.00	
Cost of unintended pregnancy, €	1,630.10	1,329.96	1,802.54	

*ulipristal acetate minimum price according to HRA Market research.

A cost-effectiveness analysis was performed. The results are presented first in the number of unintended pregnancies that could be avoided with the use of ulipristal acetate instead of levonorgestrel. Second, results are presented in a cost estimate of each unintended pregnancy, considering the weighted average costs of voluntary termination, miscarriage, and term delivery. Third, the costs of avoiding an unintended pregnancy with ulipristal acetate and with levonorgestrel are presented. Incremental cost-effectiveness ratio was also calculated. The following formula shows the calculation of incremental cost-effectiveness ratio for ulipristal acetate compared to levonorgestrel:

$$\begin{array}{l} Incremental\;\text{cost}-\text{effectiveness ratio}:\\ ({\text{Cost}}_{ULIPRISTAL\;ACETATE}-Cost_{levonorgestrel})/\\ \left([1-pregnancy\;rate_{ULIPRISTAL\;ACETATE}]-[1-pregnancy\;rate_{levonorgestrel}]\right) \end{array}$$

This ratio therefore defines the incremental cost that must be paid to avoid one additional pregnancy.

Ulipristal acetate is considered to be cost-effective if this ratio is found to be below the cost of an unintended pregnancy. The base case analysis considers the mean estimates of probabilities and costs. To delineate the robustness of the analysis, extreme values are used in sensitivity analyses. Further analyses consider the pregnancy rate of emergency contraception when used within 24 hours after unprotected intercourse.

Economic analyses are based on assumptions. Consequently, they are characterized by uncertainty. Sensitivity analyses aim to test the robustness of the base case analysis by varying the most uncertain parameters. For this analysis, deterministic (univariate and two-way) and probabilistic sensitivity analyses were performed. In the univariate deterministic analyses, parameters are varied one by one (ulipristal acetate and levonorgestrel product costs and voluntary termination and miscarriage costs, as well as pregnancy outcome probabilities) by maintaining all other factors identical to the base case. In the two-way analysis, two parameters are varied at the same time. The most sensitive parameters were chosen for variation.

## Results

In 2010, the number of unintended pregnancies in minors that could have been avoided with the use of ulipristal acetate instead of levonorgestrel is estimated to be 2,862 (range 2,355–7,608) (Table 4). The cost of an unintended pregnancy in a French minor is calculated to be 1,630 € (1,330 €–1,803 €) (Table 3). With these estimates, 3.9 million € (3.1 €–13.7 € million) of unintended pregnancy costs could have been saved by using ulipristal acetate instead of levonorgestrel in 2010. Increasing the rate of emergency contraception use increases the potential savings. The incremental cost of ulipristal acetate compared to levonorgestrel is 3.30 € at an incremental effectiveness of 0.79%. This leads to an incremental cost-effectiveness ratio of 418 € for emergency contraception intake within 72 hours after unprotected intercourse (Table 5).

**Table 4:** Number of pregnancies avoided. Range uses 95% confidence intervals of pregnancy rates.

	Number of unintended pregnancies avoided/ 1,000 emergency contraception users			
Emergency contraception intake	Levon- orgestrel	Ulipristal acetate	Additional pregnancies avoided with ulipristal acetate	Potential further pregnancies avoided in minors in 2010
Within 72 hours,	**35**	**43**	**8**	**2,862**
**n** (range)	(27–41)	(36–48)	(7–9)	(2,355–3,369)
Within 24 hours,	**31**	**48**	**17**	**5,978**
**n** (range)	(16–42)	(37–54)	(11–21)	(4,094–7,608)

* In emergency contraception users < 18 years.

**Table 5:** Incremental cost and effectiveness of ulipristal acetate versus levonorgestrel. UPA dominant, more effective at lower cost;

	UPA,	LNG	Difference (UPA-LNG)	Incremental cost per pregnancy avoided (ICER)	
**Base case (intake 0–72 hours)**					
Cost of unintended pregnancy per intake, €*	22.17	35.05			
Drug costs, €	23.59	7.41			
Cost per patient, €	45.76	42.46	3.30		
Pregnancy rate, %	1.36	2.15	0.79	418.00	
**Subgroup (intake 0–24 hours)**					
Cost of unintended pregnancy per intake, €*	13.86	40.75			
Drug costs, €	23.59	7.41			
Cost per patient, €	37.45	48.16	-10.72		
Pregnancy rate, %	0.85	2.50	1.65	-649.49	UPA dominant

* The cost of unintended pregnancy per intake of UPA or LNG was calculated by multiplying the costs of an unintended pregnancy (1,630 €) with pregnancy rates observed for each of the drugs.

Sensitivity analyses show that the results are robust. In the univariate analyses (Table 6), ulipristal acetate is the dominant method in the base case at a cost of 19.90 €, meaning that it is more effective at a lower cost. In the intake within 24 hours subgroup, ulipristal acetate is the dominant method in 5 out of 6 analyses.

**Table 6:** Univariate sensitivity analysis of the cost-effectiveness results of levonorgestrel vs ulipristal acetate to variations in main variables of the base case and subgroup models. UPA, ulipristal acetate; LNG, levonorgestrel; UPA dominant, more effective at lower cost; pab, probability of abortion; pdel, probability of delivery; pmis, probability of miscarriage after unprotected intercourse.

		Base case (intake within 72 hours)			Subgroup (intake within 24 hours)		
Variable	Variable range (low–high)	Incremental cost per patient, € (low–high)	Incremental cost per pregnancy avoided (ICER), € (low–high)		Incremental cost per patient, € (low–high)	Incremental cost per pregnancy avoided (ICER), € (low–high)	
Cost of UPA, €	19.9 to 23.59	-0.39 to 3.3	-49.08 to 418.00	UPA dominant at low value	-14.41 to -10.72	-873.13 to -649.49	UPA dominant at both values
Cost of LNG, €	4.00 to 7.41	6.71 to 3.30	849.65 to 418.00		-7.31 to 10.72	-442.82 to -649.49	UPA dominant at both values
Cost of birth, €	3733 to 5024	5.22 to 2.39	660.57 to 303.10		-6,71 to -12.61	-406.92 to -764.39	UPA dominant at both values
Cost of miscarriage, €	664 to 791	3.32 to 3.28	420.24 to 415.80		-10.68 to -0.75	-647.25 to -651.70	UPA dominant at both values
Cost of termination, €	396.23 to 557.08	3.74 to 2.87	473.33 to 362.67		-9.80 to -11.63	-594.16 to -704.83	UPA dominant at both values
Pregnancy outcome	pab: 0.723 to 0.713 pdel: 0.085 to 0.287 pmis: 0.192 to 0.0	9.26 to 3.05	1,171.95 to 385.48		1.72 to -11.25	104.45 to -682.02	UPA dominant at high values

**Fig 2:**
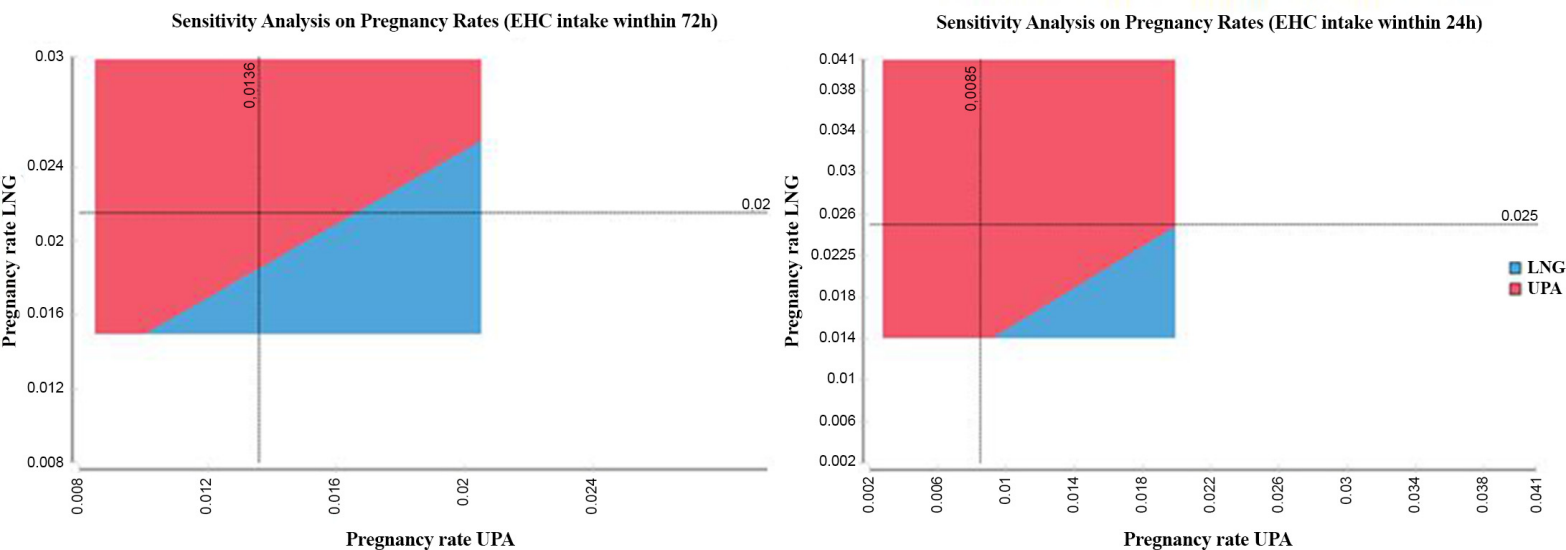
Two-way sensitivity analysis of the pregnancy rates of ulipristal acetate and levonorgestrel. UPA, ulipristal acetate; LNG, levonorgestrel; EHC, emergency hormonal contraception; Note: The colored area indicates which method is more cost-effective at a given pregnancy rate of UPA and LNG (for instance UPA is more cost-effective when the pregnancy rate of UPA is less than 1.36% and the pregnancy rate of LNG is more than 2.15% at intake within 72 hours). The dotted lines indicate the pregnancy rates observed in clinical trials, the whole area covers the 95% confidence intervals.

In the two-way analysis, two parameters are varied at the same time. The pregnancy rates of both drugs are varied, using confidence intervals as indicated in Table 1. Fig 2 shows that ulipristal acetate remains the dominant method in the majority of cases.

**Fig 3:**
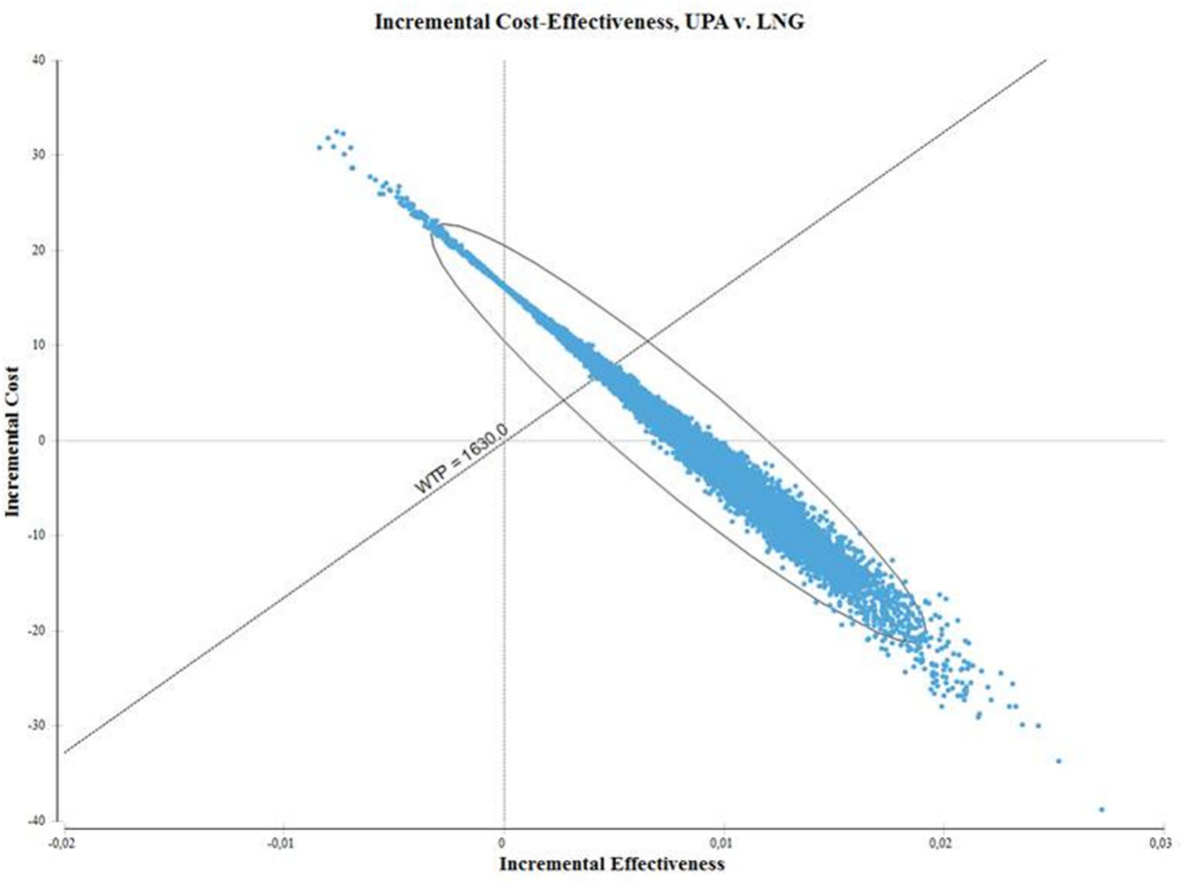
Cost-effectiveness plane of ulipristal acetate versus levonorgestrel (intake within 72 hours). WTP, willingess-to-pay, defined as the cost of an unintended pregnancy; UPA, ulipristal acetate; LNG, levonorgestrel. Note: in the upper-right quadrant, UPA costs more and is more effective than LNG; in the lower-right quadrant, UPA costs less and is more effective. UPA is cost-effective for all iterations below the WTP threshold.

The result of the probabilistic sensitivity analysis is presented in the form of a cost-effectiveness plane in Fig 3. The majority of the results after 10,000 iterations were below the willingness-to-pay threshold of 1,630.10 € (the cost of an unintended pregnancy), indicating that the results are robust. Ulipristal acetate was the preferred method in 76.9% of cases and was found to be superior to levonorgestrel in 45%, meaning it was more effective at a lower cost.

## Discussion

Ulipristal acetate has several advantages over levonorgestrel. First, it is approved for use during a larger time window than is levonorgestrel. In clinical trials, 34% of women took emergency contraception within the first 24 hours of unprotected intercourse [20]. In practice, 88% of women take emergency contraception within 24 hours after unprotected intercourse [32]. The second advantage over levonorgestrel is much more important: ulipristal acetate has been shown to significantly decrease the risk of pregnancy after unprotected intercourse compared to levonorgestrel [20]. However, the cost of ulipristal acetate is higher than that of levonorgestrel. In this research, it is calculated that the additional costs paid to avoid one additional pregnancy with ulipristal acetate compared to levonorgestrel are worth paying, because it is still less than the cost of an unintended pregnancy. Ulipristal acetate has the potential to prevent more unintended pregnancies than does levonorgestrel, a benefit that outweighs drug expenditures and can generate cost savings to health insurers. This analysis is most sensitive to the drug costs and pregnancy rates observed. Some authors criticize the claim of cost savings or cost-effectiveness of emergency contraception because a significant reduction of unintended pregnancy rates has yet to be observed on a population level [38]. However, it must be noted that this analysis uses the standard approach to evaluate cost-effectiveness. Similarly to other interventions in which cost-effectiveness has been evaluated, it assumes that emergency contraception has to be taken appropriately to work effectively. Most likely, barriers to the use of emergency contraception such as limited risk awareness [8] lead to an under-utilization that masks the impact of emergency contraception in the real world.

This analysis has some limitations. First, the cost-effectiveness analysis presents the results as cost per pregnancy avoided. It does not include quality of life. This limits its comparability to other health care interventions. Although health-related quality of life is important for unintended pregnancy, especially when experienced at very young age, only one study measured the health utility of women experiencing an unintended pregnancy [39]. The time that women will remain in this health state has not been measured. Bayer et al. [25] used the utility identified by Schwarz et al. [39] in their cost-utility analysis, which assumes that women will remain in this health state during their entire remaining lifespan. This approach overestimates the impact of unintended pregnancy on women’s health. Other economic evaluations for emergency contraception as well as for contraceptive methods also calculated the cost per pregnancy avoided [12,17,23,24]. Second, its estimate of the cost of pregnancy may be imprecise. One could argue that the estimate is an overstatement of the real cost to the community. Pregnancies that could be avoided today may occur later, i.e. are only mistimed, and the cost is not prevented but delayed [40]. However, this is not relevant to this analysis, as the target population is aged 15–17 years, whereas the mean age at first birth in France is 28 years [41]. The cost of pregnancies occurring at that age can be ignored today. In addition, it is assumed in this analysis that miscarriages require a hospital stay. However, many miscarriages do not require hospital stay [42] and costs related to these miscarriages may therefore be lower. This is investigated in the sensitivity analysis, in which it is assumed that the miscarriage rate is 0. With respect to the rate of miscarriage, it must be noted that the incidence of miscarriage in the general population is estimated to be higher [42–45] than that used in the base case. This can be explained by the fact that the risk of miscarriage increases with the age of the patient [46,47]. One could also argue that the cost of pregnancy is under-estimated in our analysis. It is acknowledged that the amounts used for the calculation of the costs of unintended pregnancy in this analysis are not specific to minors, but are based on the cost of pregnancy for all women. Although applying the same costs specifically to minors may not influence the result, it neglects the social and psychological impact of an unintended pregnancy on the life of a patient in this age group. Consequently, the negative impact of unintended pregnancy in minors can be estimated to be higher than in the general population, thus increasing the cost of pregnancy. However, this impact is difficult to quantify. In addition, we have used standard tariffs for the calculation of costs of pre- and postnatal care in outpatients. Specialists can charge more than these tariffs, and it has been shown that gynecologists in sector 2 charge an average of 60% more than the standard tariff [48]. Finally, the time horizon of this analysis is limited to some days after birth. There may be costs beyond the end of the termination procedure, and the real costs of live birth are higher–childcare and healthcare for the mother are especially expensive in the first year after birth. Moreover, governmental financial assistance is provided to families in France; so-called “parent money”. In addition to the assistance prior to delivery that is considered in this analyses, these benefits include a base allocation during the first three years after delivery (monthly benefits for a couple without revenue are estimated to be 185 €, adding up to about 6,660 € in total over three years [49]). Underestimation of the cost of unintended pregnancy leads to an underestimation of the cost-effectiveness of ulipristal acetate compared to levonorgestrel. In total, the approach to defining and valuing resource utilization in this analysis can be considered to be conservative, and presents rather an underestimation of the costs of unintended pregnancy in minors. Third, pregnancy rates used in this analysis come from clinical trials where condition of use may differ from real life. According to a recent review, perfect utilization may not be the general rule for emergency contraception [8]. However, pregnancy trials performed to evaluate the efficacy and safety of ulipristal acetate as emergency contraceptive were conducted in situations very close to real life use. In addition, the safety and efficacy of ulipristal acetate for emergency contraception has recently been evaluated in adolescent and adult women in a large observational study. The results of this study confirm that efficacy and safety profiles of ulipristal acetate are consistent with those observed in clinical trials, and there was no difference in efficacy or safety between adolescent and adult women [50]. It must also be mentioned that we used the number of emergency contraception intakes that occurred in 2010 to calculate the number of pregnancies that can be avoided by using ulipristal acetate compared to levonorgestrel. However, the annual intake of emergency contraception reimbursed for minors increased by 18.6% from 2009–2010 [8]. No figures for later years are available, but it can be expected that increasing numbers of minors have used emergency contraception in subsequent years, which increases the annual number of unintended pregnancies that can be avoided. Interestingly, the total emergency contraception market was almost stable over the same period [51]. Fourth, ulipristal acetate is assumed to be superior to levonorgestrel in decreasing the risk of pregnancy after unprotected intercourse. This is based on the meta-analysis of Glasier et al. [20]. Cochrane [52] performed their own meta-analysis based on the same clinical trials as those used in the meta-analysis. Cochrane Reviews have an international reputation of providing the highest standard in evidence-based health care. The Cochrane review concluded that “ulipristal acetate appeared more effective than levonorgestrel at a marginal level (10%)” rather than at the 5% level as found in the analysis of Glasier et al. [20]. It should be mentioned that the methodology used in the Glasier study took into account confounding factors for pregnancy risk (further intercourse, conception probability, and body mass index) in the estimation of treatment effect. This was not the case in the Cochrane review. The different ways of combining the trials do not have an effect on the observed pregnancy rates used in the present analysis. While the use of odds ratios/risk ratios is common in meta-analyses, it is more common and generally advisable to collect raw data on outcome measures, such as numbers of people treated, rather than using derived measures such as odds ratios in economic analyses [53]. Moreover, the effect of changing pregnancy rates is analyzed in the two-way sensitivity analysis.

It is also worthwhile to discuss the strengths of the analysis. Pregnancy rates were taken from head-to-head randomized clinical trials. It was also possible to calculate the 95% confidence intervals of the combined pregnancy rates, which increases the robustness of the findings. In addition, no economic evaluation has been published so far on the use of emergency contraception in French minors. Finally, with the help of the subgroup analyses, this research allows for comparison of the cost-effectiveness of ulipristal acetate compared to levonorgestrel within different time windows of emergency contraception intake. Because 88% of French women use emergency contraception within the first 24 hours after unprotected intercourse [32], this subgroup analyses is important in practice. In this subgroup, ulipristal acetate dominates levonorgestrel in almost all cases.

The conclusions of this analysis are consistent with the conclusions of other analyses comparing the use of ulipristal acetate and levonorgestrel as emergency contraception, even when the cost of an unintended pregnancy varies from one country to another. In the UK, Thomas et al. [23] conclude that ulipristal acetate is cost-effective compared to levonorgestrel because the cost per pregnancy avoided (reference case cost 311 £, ranging from 183 £–500 £) is below the cost of an unintended pregnancy in the UK (948 £). Rubio-Terrés et al. [24] estimated that the cost of avoiding an additional pregnancy with ulipristal acetate compared to levonorgestrel is 108 €, ranging from 3 € to 567 € in sensitivity analyses. In 37% of the sensitivity analyses, ulipristal acetate dominated levonorgestrel, meaning that it was more effective at a lower cost. The cost of an unintended pregnancy in Spain was estimated at 1,671 €. Bayer at al. [25] found that ulipristal acetate would be cost-effective up to a price of 265 $, which is far higher than the current price of ulipristal acetate.

Unintended pregnancy among adolescents can be reduced with a combination of educational and contraceptive interventions [54]. Easy access to emergency contraception can decrease the risk of pregnancy. Even though it must be acknowledged that the interventions tested so far have not reduced pregnancy rates [55] because of high rates of unprotected intercourse and relative underutilization [56], facilitating access to emergency contraception among adolescents increases usage without compromising the use of regular contraception or increasing risky sexual behavior [57]. Efforts by the French government to increase the accessibility of (emergency) contraception have shown positive results during recent years. The utilization of emergency contraception has increased considerably over the last decade and is highest among minors. Nonetheless, utilization remains low compared to the situations at risk. Over-the-counter access to ulipristal acetate is crucial to facilitating the availability of this cost-effective method to minors. Information about situations at risk and appropriate emergency contraception use has to be improved further to benefit fully from the cost-effectiveness of ulipristal acetate. These measures should help to further increase the utilization of emergency contraception.

In conclusion, this study is the first economic analysis to consider emergency contraception delivery and unintended pregnancy outcomes among minors in France. The results of the study demonstrate that ulipristal acetate dominates levonorgestrel when taken within 24 hours after unprotected intercourse, i.e., it is more effective at a lower cost. When taken within 72 hours, ulipristal acetate is a cost-effective alternative to levonorgestrel given that the cost of avoiding an additional pregnancy with ulipristal acetate is less than the average cost of these pregnancies. Future studies that examine the impact of emergency contraception on unintended pregnancy rates in minors will help follow the impact of emergency contraception on a population level.

## Supporting Information

S1 File 95% confidence interval calculations. Any queries about the original data can be sent to the author.
(DOCX)

S2 File Summary data from Agence Technique de l’Information sur l’Hospitalisation (ATIH). Guide de l’étude nationale de coûts à méthodologie commune–MCO [30].
(XLSX)
