# Supplementary material for: The Cost-Effectiveness of Emergency Hormonal Contraception with Ulipristal Acetate versus Levonorgestrel for Minors in France
Source: PLoS One. 2015 Sep 30;10(9):e0138990. doi: 10.1371/journal.pone.0138990 (PMC4589416; doi:10.1371/journal.pone.0138990)
Supplement: S1 File — Any queries about the original data can be sent to the author. (DOCX) [file pone.0138990.s001.docx]

**95% Confidence Interval calculation**

**Method**: Clopper Pearson method (exact confidence interval).

04/05/13

**For HRA**

Bruno Scherrer

|  | Pregnancies* | | | |
| --- | --- | --- | --- | --- |
|  | Ulipristal acetate | | Levonorgestrel | |
|  | Point Estimate | Exact 95% C.I. | Point estimate | Exact 95% C.I. |
| Creinin et al.  0-72h | 0.91 % (7/773) | 0.36%, 1.86% | 1.68 % (13/773) | 0.90%, 2.86% |
| Current study  0 – 120 h. | 1.59 % (15/941) | 0.89%, 2.62% | 2.61% (25/958) | 1.70%, 3.83% |
| Meta-analyse  0-24h | 0.86 % (5/584) | 0.28%, 1.99% | 2.50% (15/600) | 1.41%, 4.09% |
| Meta-analyse  0 – 72 h | 1.36% (22/1617) | 0.85%, 2.05% | 2.15% (35/1625) | 1.50%, 2.98% |
| Meta-analyse  0 – 120 h | 1.28% (22/1714) | 0.81%, 1.94% | 2.20% (38/1731) | 1.56%, 3.00% |

* tableau vérifié par rapport au tableau ci-dessous et aux outputs (en fin de document)


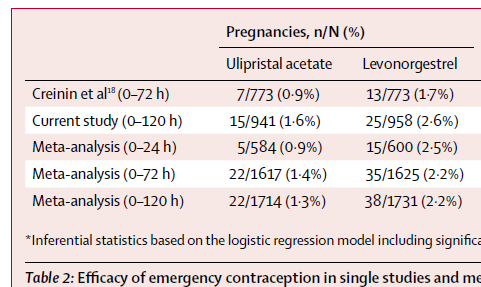


!Cytel Studio (9.0.0)

(Mar 17,2010)

Date: Saturday, May 4, 2013

Time: 6:39:46

Note: StatXact-9 (9.0.0) is licensed to Bruno Scherrer Conseil

Site: Cytel Inc Pune (Test License) ( 2099263 )

Expiry Date: No expiration.

Installed Path: C:\Program Files\Cytel Studio 9\StatXact

GLOBAL OPTIONS

Input Accuracy: 3

Output Display: Auto

FILE PATHS

Data Files: C:\Documents and Settings\Scherrer Bruno\Mes documents\My Cytel Files\9.0

Submit Files: C:\Documents and Settings\Scherrer Bruno\Mes documents\My Cytel Files\9.0

Exact Distribution Files: C:\Documents and Settings\Scherrer Bruno\Mes documents\My Cytel Files\9.0

Output Files: C:\Documents and Settings\Scherrer Bruno\Mes documents\My Cytel Files\9.0

Workbook Files: C:\Documents and Settings\Scherrer Bruno\Mes documents\My Cytel Files\9.0

Temporary Files: C:\DOCUME~1\SCHERR~1\LOCALS~1\Temp\

ESTIMATION OF BINOMIAL PARAMETER (PI)

Number of Trials =773

Number of Successes =7

Maximum Likelihood Estimate of PI = 0.0091

95.00% Confidence Interval for PI:

(Clopper-Pearson) = (0.0036 ,0.0186)

Exact P-values for testing PI = 0.5000

One-sided : Pr { T .LE. 7 } = 0.0000

Pr { T .EQ. 7 } = 0.0000

Two-sided : 2 * One-sided = 0.0000

Elapsed Time is 0:0:0.03

ESTIMATION OF BINOMIAL PARAMETER (PI)

Number of Trials =773

Number of Successes =13

Maximum Likelihood Estimate of PI = 0.0168

95.00% Confidence Interval for PI:

(Clopper-Pearson) = (0.0090 ,0.0286)

Exact P-values for testing PI = 0.5000

One-sided : Pr { T .LE. 13 } = 0.0000

Pr { T .EQ. 13 } = 0.0000

Two-sided : 2 * One-sided = 0.0000

Elapsed Time is 0:0:0.05

ESTIMATION OF BINOMIAL PARAMETER (PI)

Number of Trials =941

Number of Successes =15

Maximum Likelihood Estimate of PI = 0.0159

95.00% Confidence Interval for PI:

(Clopper-Pearson) = (0.0089,0.0262)

Exact P-values for testing PI= 0.5000

One-sided : Pr { T .LE. 15 } = 0.0000

Pr { T .EQ. 15 } = 0.0000

Two-sided : 2 * One-sided = 0.0000

Elapsed Time is 0:0:0.05

ESTIMATION OF BINOMIAL PARAMETER (PI)

Number of Trials =958

Number of Successes =25

Maximum Likelihood Estimate of PI= 0.0261

95.00% Confidence Interval for PI:

(Clopper-Pearson) = (0.0170,0.0383)

Exact P-values for testing PI= 0.5000

One-sided : Pr { T .LE. 25 } = 0.0000

Pr { T .EQ. 25 } = 0.0000

Two-sided : 2 * One-sided = 0.0000

Elapsed Time is 0:0:0.02

ESTIMATION OF BINOMIAL PARAMETER (PI)

Number of Trials =584

Number of Successes =5

Maximum Likelihood Estimate of PI = 0.0086

95.00% Confidence Interval for PI:

(Clopper-Pearson) = (0.0028,0.0199)

Exact P-values for testing PI= 0.5000

One-sided : Pr { T .LE. 5 } = 0.0000

Pr { T .EQ. 5 } = 0.0000

Two-sided : 2 * One-sided = 0.0000

Elapsed Time is 0:0:0.02

ESTIMATION OF BINOMIAL PARAMETER (PI)

Number of Trials =600

Number of Successes =15

Maximum Likelihood Estimate of PI = 0.0250

95.00% Confidence Interval for PI:

(Clopper-Pearson) = (0.0141, 0.0409)

Exact P-values for testing PI= 0.5000

One-sided : Pr { T .LE. 15 } = 0.0000

Pr { T .EQ. 15 } = 0.0000

Two-sided : 2 * One-sided = 0.0000

Elapsed Time is 0:0:0.03

ESTIMATION OF BINOMIAL PARAMETER (PI)

Number of Trials =1617

Number of Successes =22

Maximum Likelihood Estimate of PI = 0.0136

95.00% Confidence Interval for PI:

(Clopper-Pearson) = (0.0085,0.0205)

Exact P-values for testing PI= 0.5000

One-sided : Pr { T .LE. 22 } = 0.0000

Pr { T .EQ. 22 } = 0.0000

Two-sided : 2 * One-sided = 0.0000

Elapsed Time is 0:0:0.08

ESTIMATION OF BINOMIAL PARAMETER (PI)

Number of Trials =1625

Number of Successes =35

Maximum Likelihood Estimate of PI = 0.0215

95.00% Confidence Interval for PI:

(Clopper-Pearson) = (0.0150,0.0298)

Exact P-values for testing PI= 0.5000

One-sided : Pr { T .LE. 35 } = 0.0000

Pr { T .EQ. 35 } = 0.0000

Two-sided : 2 * One-sided = 0.0000

Elapsed Time is 0:0:0.09

ESTIMATION OF BINOMIAL PARAMETER (PI)

Number of Trials =1714

Number of Successes =22

Maximum Likelihood Estimate of PI = 0.0128

95.00% Confidence Interval for PI:

(Clopper-Pearson) = (0.0081,0.0194)

Exact P-values for testing PI= 0.5000

One-sided : Pr { T .LE. 22 } = 0.0000

Pr { T .EQ. 22 } = 0.0000

Two-sided : 2 * One-sided = 0.0000

Elapsed Time is 0:0:0.16

ESTIMATION OF BINOMIAL PARAMETER (PI)

Number of Trials =1731

Number of Successes =38

Maximum Likelihood Estimate of PI = 0.0220

95.00% Confidence Interval for PI:

(Clopper-Pearson) = (0.0156,0.0300)

Exact P-values for testing PI= 0.5000

One-sided : Pr { T .LE. 38 } = 0.0000

Pr { T .EQ. 38 } = 0.0000

Two-sided : 2 * One-sided = 0.0000

Elapsed Time is 0:0:0.08
